# The Principles of Surgery

**Published:** 1852-08

**Authors:** 


*The Principles of Surgery.* By JAMES MILLER, F. R. S. E., F. R. C. S. E. &c. &c. *Third American, from the second and enlarged Edinburgh Edition. Illustrated by two hundred and forty engravings on wood. Revised with additions by* F. W. SARGENT, M. D., Member of the College of Physicians, Author of Minor Surgery, &c. Philadelphia, Blanchard & Lea, 1852.

The third American, from the second English edition of this standard work of Professor Miller, has been greatly enriched by the notes and bibliography of the edition, so as to present a resumé of all that has been added to the literature of Surgery, since the appearance of the English prototype. Dr. F. Sargent deserves the thanks of the profession for his faithful industry. Of the original work we have already, in the previous edition, expressed our opinion, and need only add our warm recommendation of it to student and practitioner.

